# Supplementary material for: “Are you listening?”: Experiences shared online by family caregivers of patients in the palliative phase during the Covid-19-pandemic
Source: PLoS One. 2024 Nov 14;19(11):e0310624. doi: 10.1371/journal.pone.0310624 (PMC11563413; doi:10.1371/journal.pone.0310624)
Supplement: S1 File — (PDF) [file pone.0310624.s001.pdf]

**Supplementary file 1: Search terms used for data collection**

| Dutch | English |
|---|---|
| Dodelijk ziek | Fatally ill |
| Dodelijke aandoening | Fatal condition |
| Einde levensfase | End-of-life stage/phase |
| Euthanasie | Euthanasia |
| Geen genezing mogelijk | No cure possible |
| Hospice | Hospice |
| Laatste levensfase | Last stage/phase of life |
| Levensbedreigende aandoening | Life-threatening condition |
| Levenseinde | End-of-life |
| Levenseindezorg | End-of-life care |
| Levensverkortende ziekte | Life-limiting illness |
| Mantelzorg | Informal caregiving |
| Mantelzorger | Family / informal caregiver |
| Ongeneeslijk ziek | Incurably ill |
| Palliatie | Palliation |
| Palliatieve fase | Palliative phase |
| Palliatieve sedatie | Palliative sedation |

| | |
|---|---|
| Palliatieve zorg | Palliative care |
| Palliative zorgverlening | Palliative care provision |
| Rouw | Grief |
| Sterfbed | Deathbed |
| Stervensbegeleiding | Dying care |
| Terminaal<br><br>Terminale | Terminal |
| Terminale fase | Terminal phase |
| Uitbehandeld | All treatment options exhausted |
| Vergevorderd stadium | Advanced stage |
