# Supplementary material for: “Are you listening?”: Experiences shared online by family caregivers of patients in the palliative phase during the Covid-19-pandemic
Source: PLoS One. 2024 Nov 14;19(11):e0310624. doi: 10.1371/journal.pone.0310624 (PMC11563413; doi:10.1371/journal.pone.0310624)
Supplement: Attachment — Submitted filename: Article review PLOS.docx [file pone.0310624.s003.docx]

Thank you for the opportunity to review this article. I found it very interesting. My comments centre around having a description of the broader Dutch Govt approach to put some of the findings in context as well as perhaps a sharper discussion integrating the speech act analysis – these are the questions I was left with after reading. There are some other smaller comments/edits. Thank you for doing this study; it is a valuable contribution.

Line 45 - Consistency in naming of pandemic

Line 59 – insert a not?

Line 58 -61 – split the sentence

Line 68 – position of adjective “other” - move to before ‘than’ - same in line 71

Line 89 – A Reference for this proper noun name of a search strategy?

Line 89 – a little description of what was happening in the Netherlands during this first wave wld be helpful. Was this a period of time chosen that reflected a specific period of time the Government approached COVID management in a particular way? Or what changed in June 2020? A bit more context would be helpful to justify this time period chosen – in the background/introduction most likely or somewhere that puts the first theme in context - - or is the situation in lines 47 – 57 Netherlands specific?

Line 157 – a timeframe-period over which this occurred?

Line 164 Prob need to put somewhere the twitter/tweet/X platform changes…

Line 244 – incorrect use of the modal auxiliary “would” – use “should” or change construction to past – “patient became infected”

Line 293 – a reference to the restrictions – would be good to have that broader context in the introduction somewhere as previously described.

Line 385 – Can this summary paragraph reflect the speech act analysis as well, rather than a broad statement

Line 425 – the new perspective by speech act I think needs stronger representation within the discussion – why so many declaratives? Was there a hegemonic situation in The Netherlands which meant explorative or directives were not welcome? Refer back to previous questions about the broader response by the Dutch Govt (lines 89 etc)

Line 467ff – In light of the previous comment, there may be scope to sharpen your discussion and recommendations - especially from the “Call to action” theme, the normative declarative, “Birth and death should be spent together!” and any related directives. See also- Collier A, Balmer D, Gilder E*,* Parke, R. Patient safety and hospital visiting at the end of life during COVID-19 restrictions in Aotearoa New Zealand: a qualitative study. *BMJ Quality & Safety* Published Online First: 14 February 2023. doi: 10.1136/bmjqs-2022-015471
